# Supplementary figures and images for: Rho-ROCK liberates sequestered claudin for rapid de novo tight junction formation
Source: eLife. 2025 Jul 24;13:RP102794. doi: 10.7554/eLife.102794 (PMC12289309; doi:10.7554/eLife.102794)

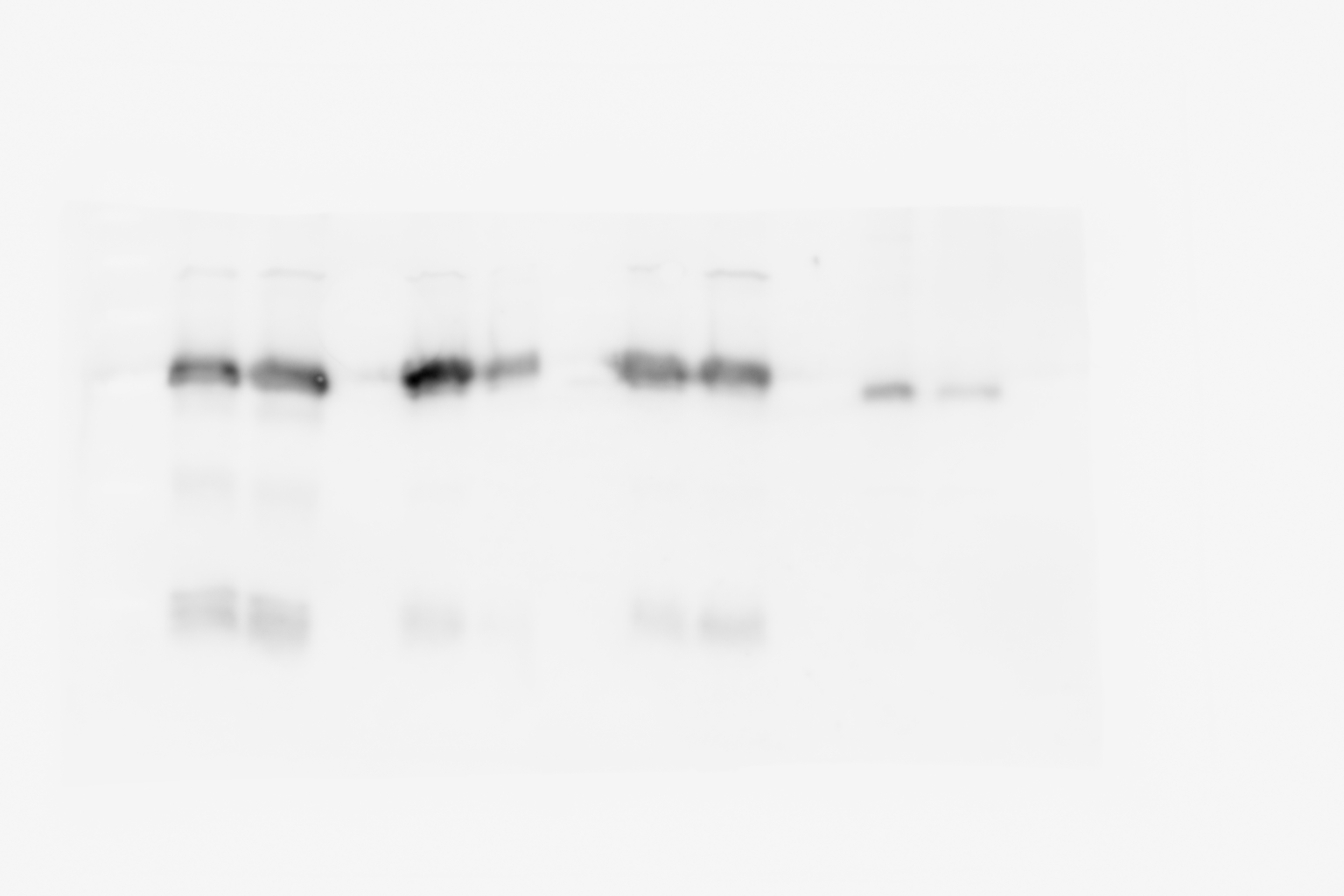

Supplement: Figure 2—source data 1. [file elife-102794-fig2-data1.zip › original images/Figure 2C anti-claudin-1.tif]

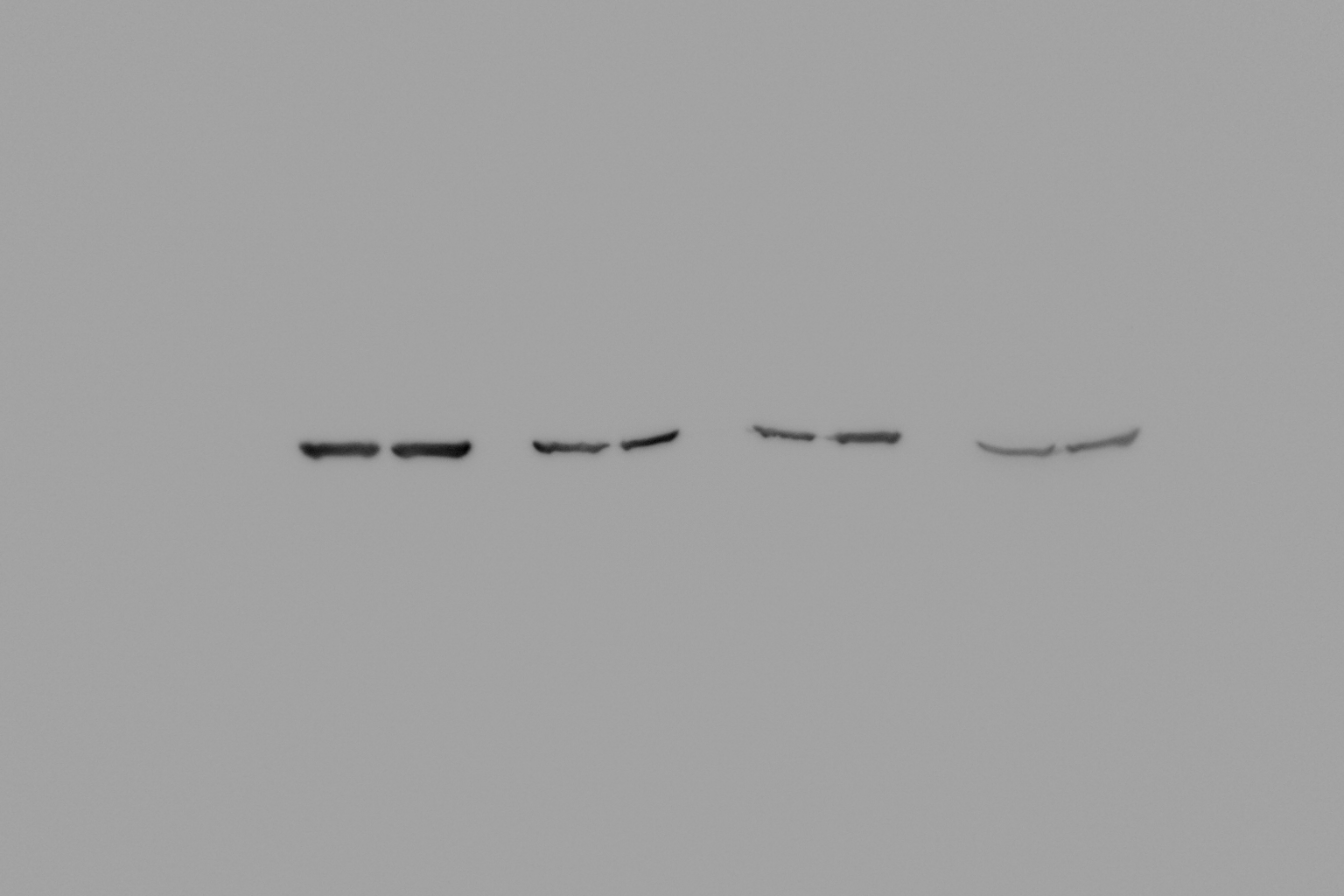

Supplement: Figure 2—source data 1. [file elife-102794-fig2-data1.zip › original images/Figure 2C anti-alpha tubulin.tif]

# Figure2C

anti-claudin-1

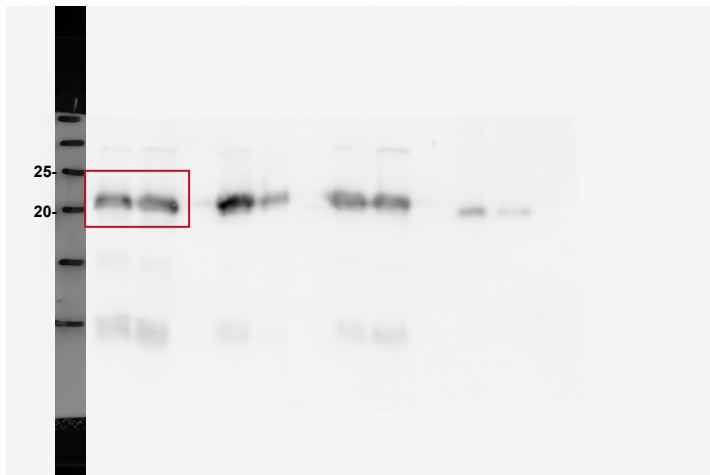

anti- $\alpha$ -tubulin

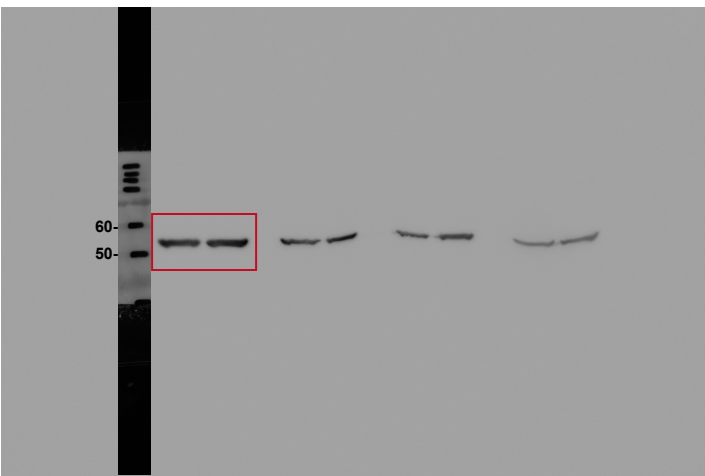

Supplement: Figure 2—source data 2. [file elife-102794-fig2-data2.pdf]

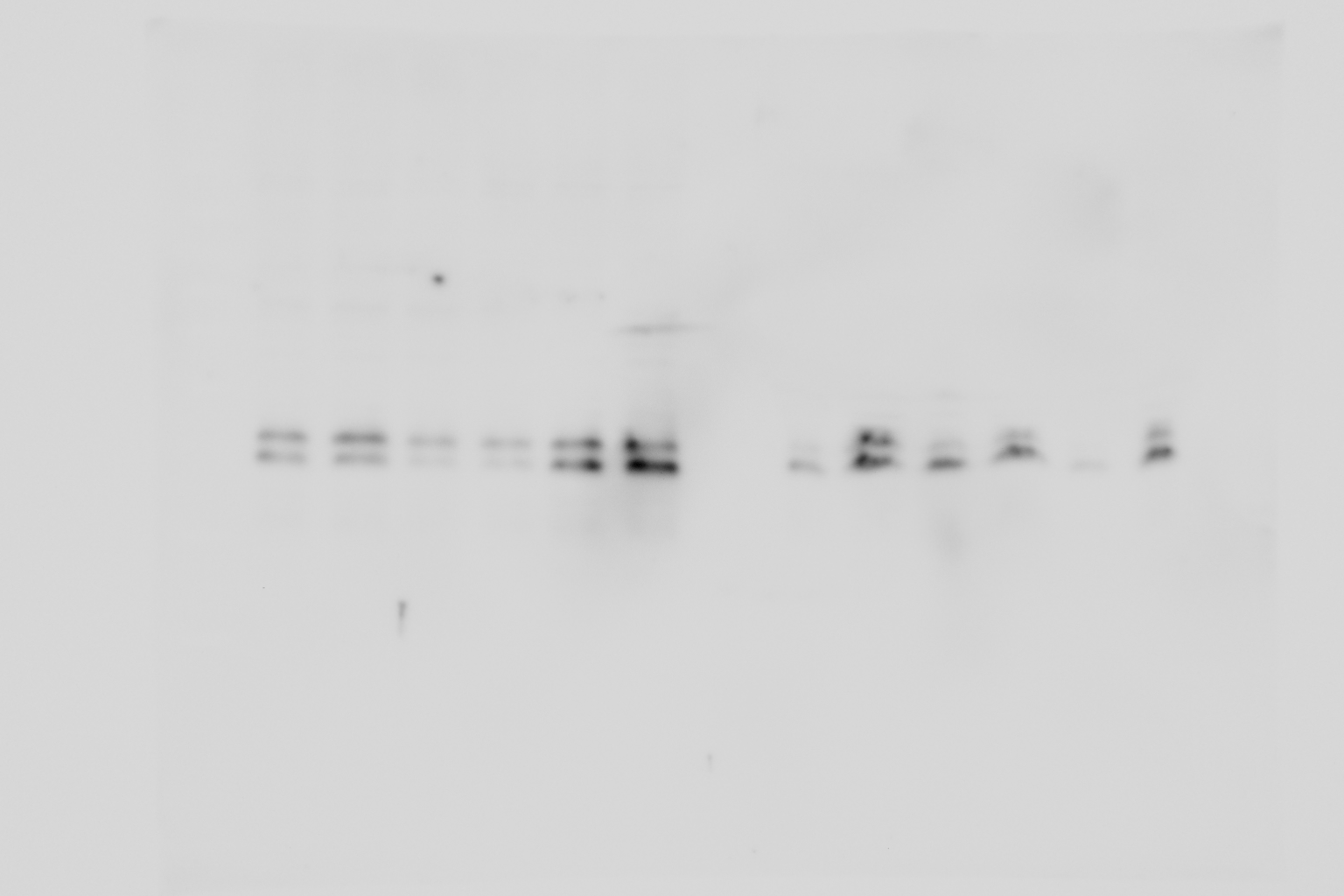

Supplement: Figure 3—source data 1. [file elife-102794-fig3-data1.zip › original images/Figure 3A anti-RhoA.tif]

# Figure3A

anti-RhoA

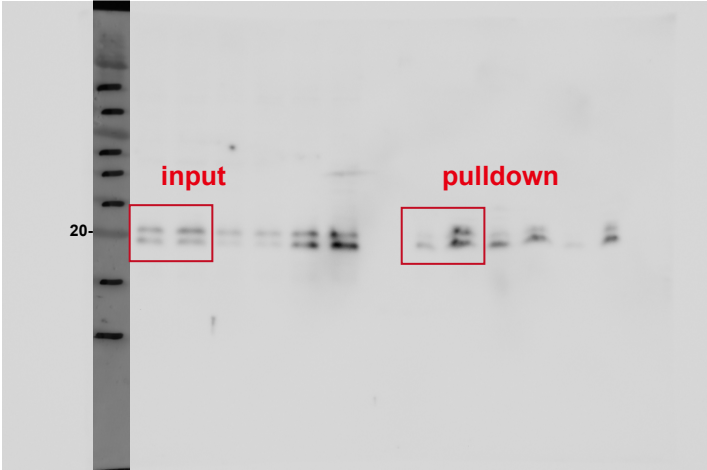

Supplement: Figure 3—source data 2. [file elife-102794-fig3-data2.pdf]

# Figure4H

anti-claudin-1

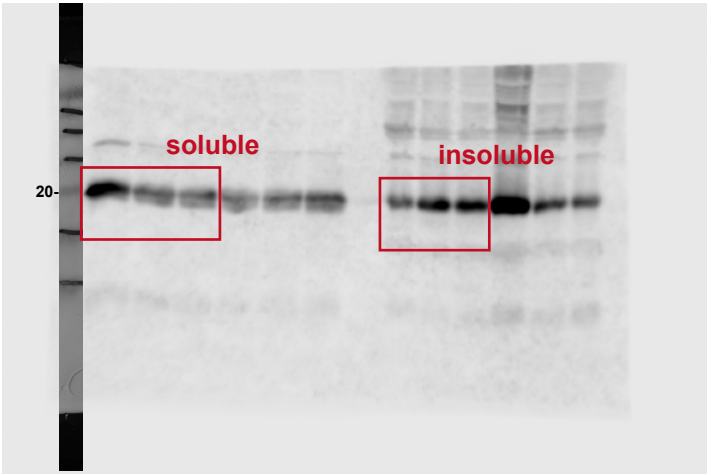

Supplement: Figure 4—source data 2. [file elife-102794-fig4-data2.pdf]

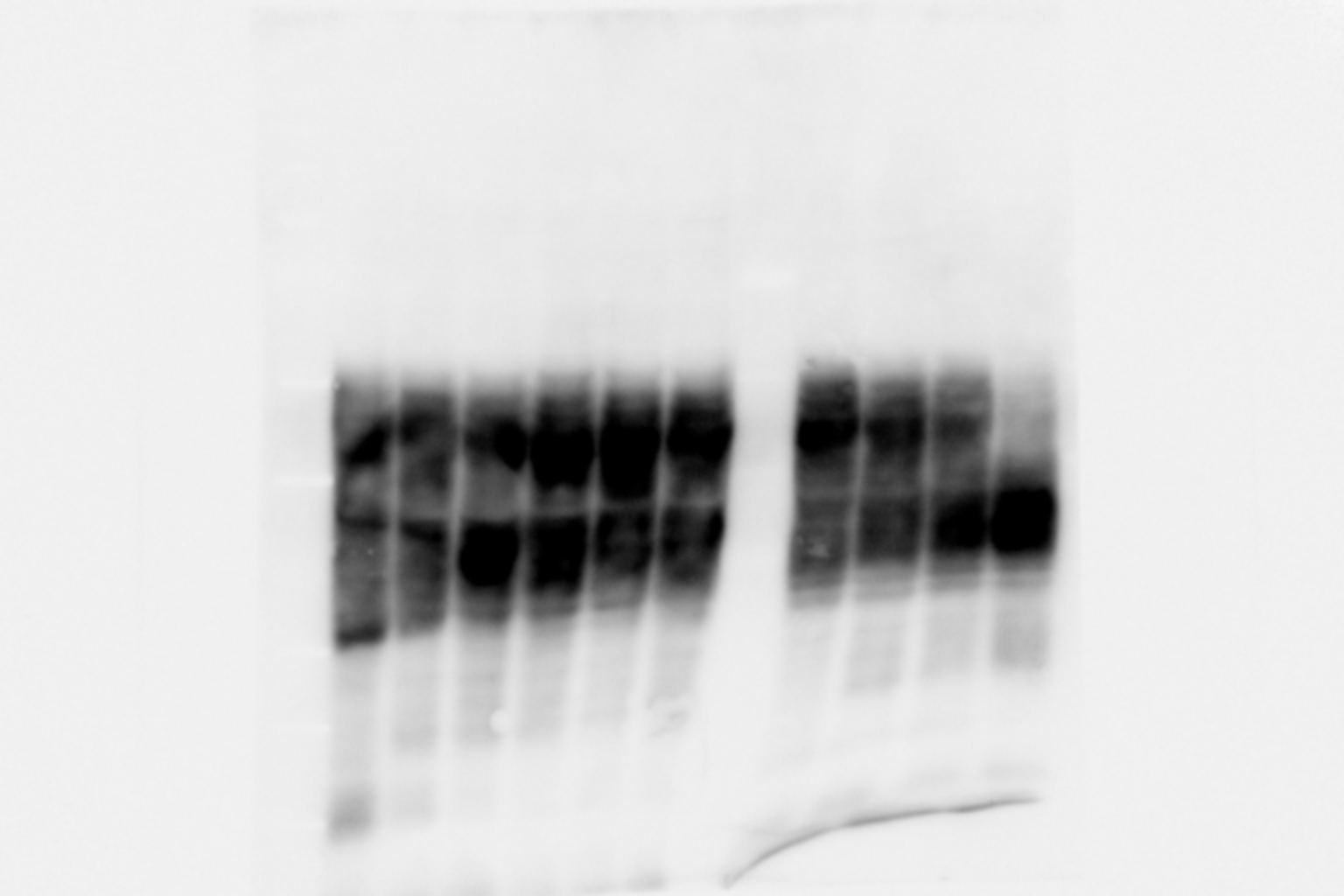

Supplement: Figure 5—source data 1. [file elife-102794-fig5-data1.zip › original images/Figure 5A anti-TROP2.tif]

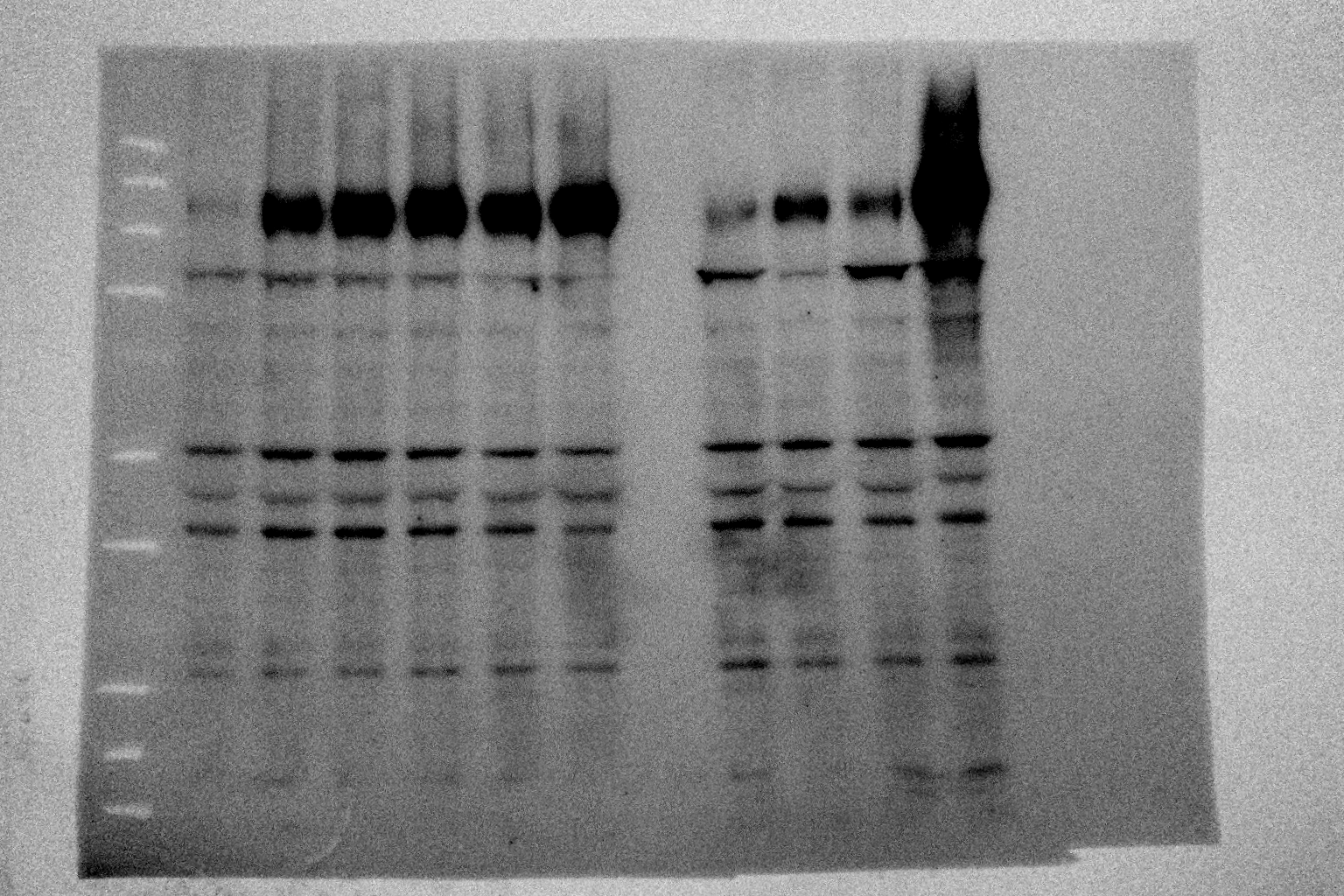

Supplement: Figure 5—source data 1. [file elife-102794-fig5-data1.zip › original images/Figure 5A anti-matriptase M69.tif]

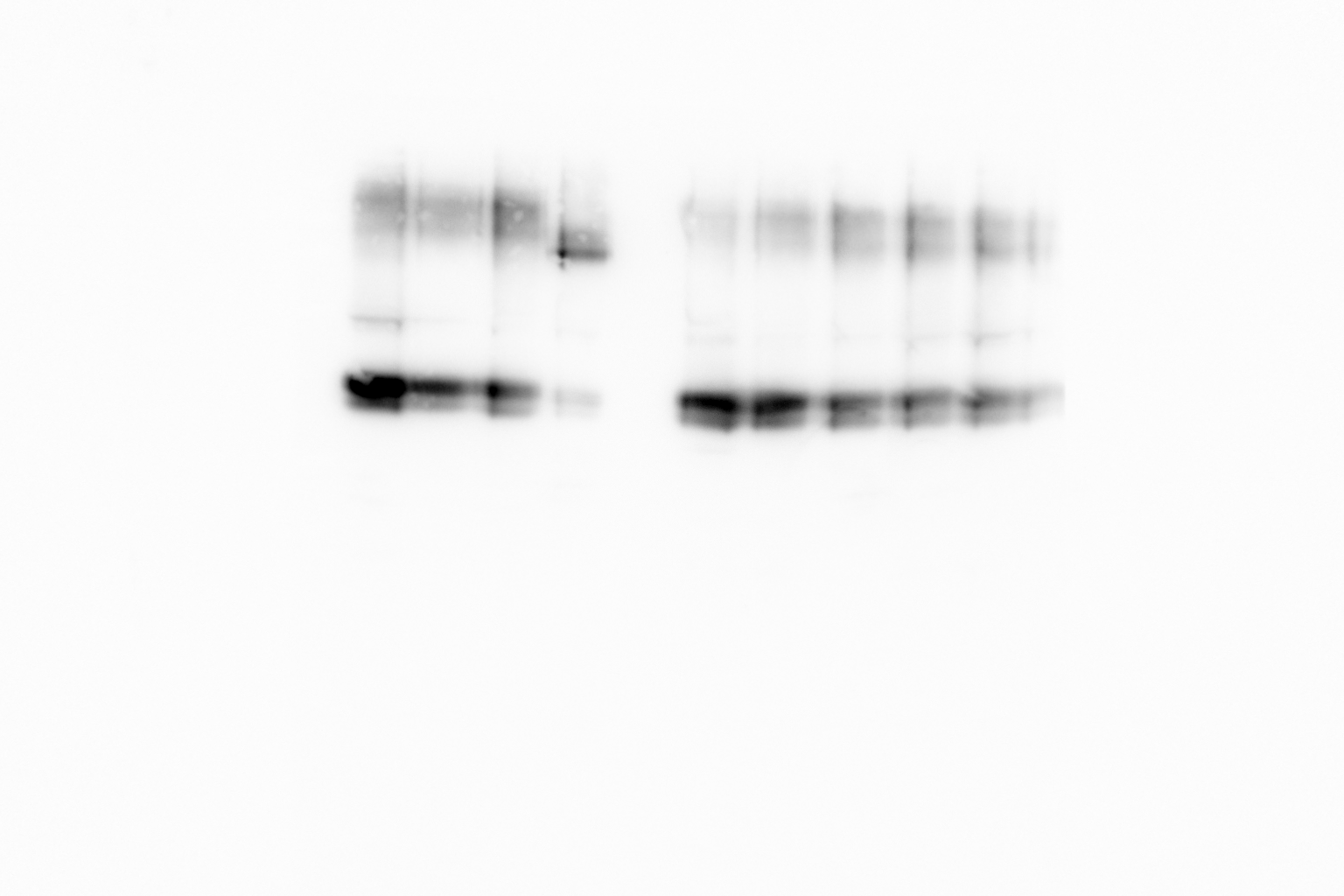

Supplement: Figure 5—source data 1. [file elife-102794-fig5-data1.zip › original images/Figure 5A anti-matriptase M24.tif]

# Figure5A and 5E

anti-Matriptase (M69)

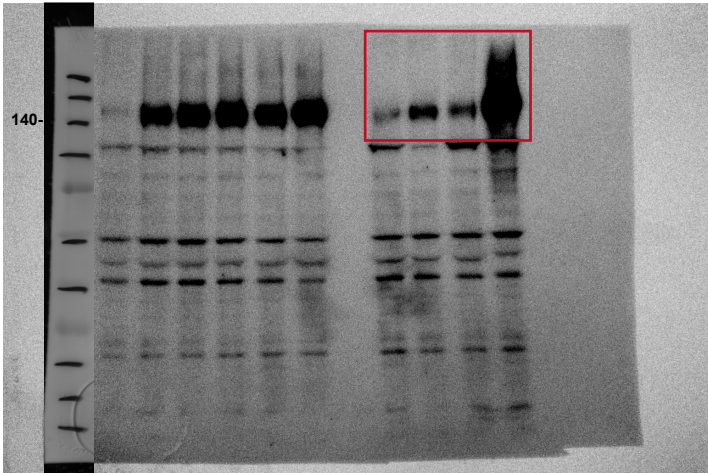

anti-claudin-1

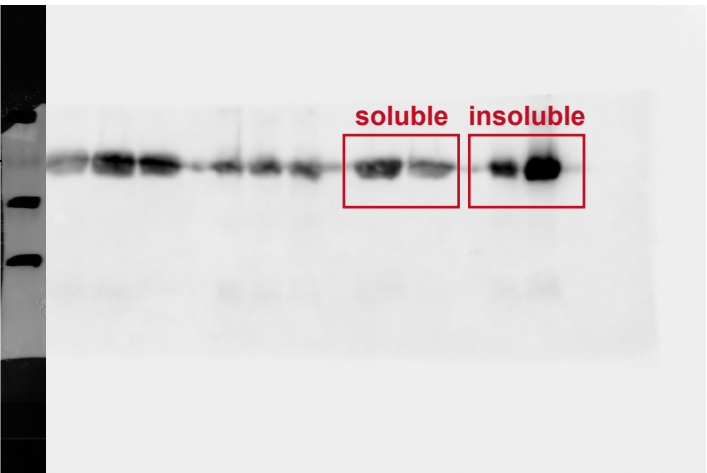

anti-Matriptase (M24)

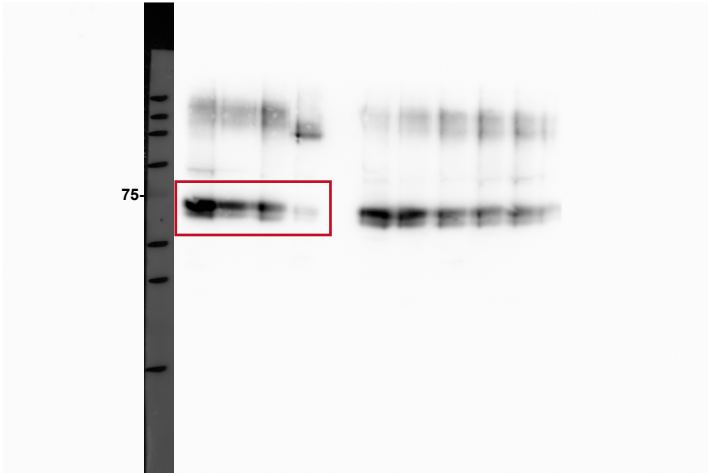

anti-TROP2

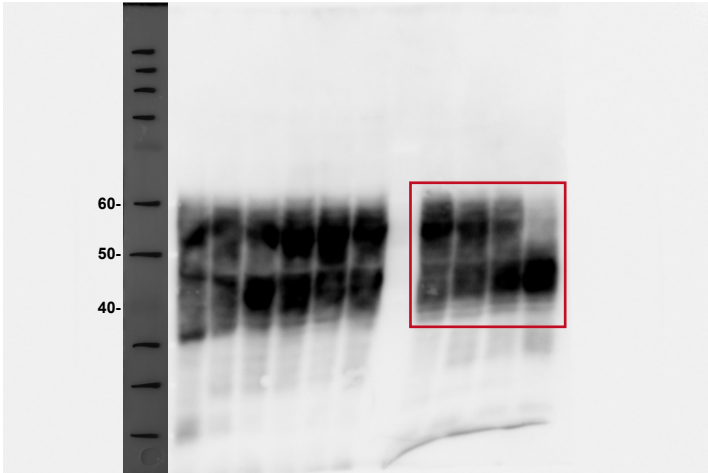

Supplement: Figure 5—source data 2. [file elife-102794-fig5-data2.pdf]

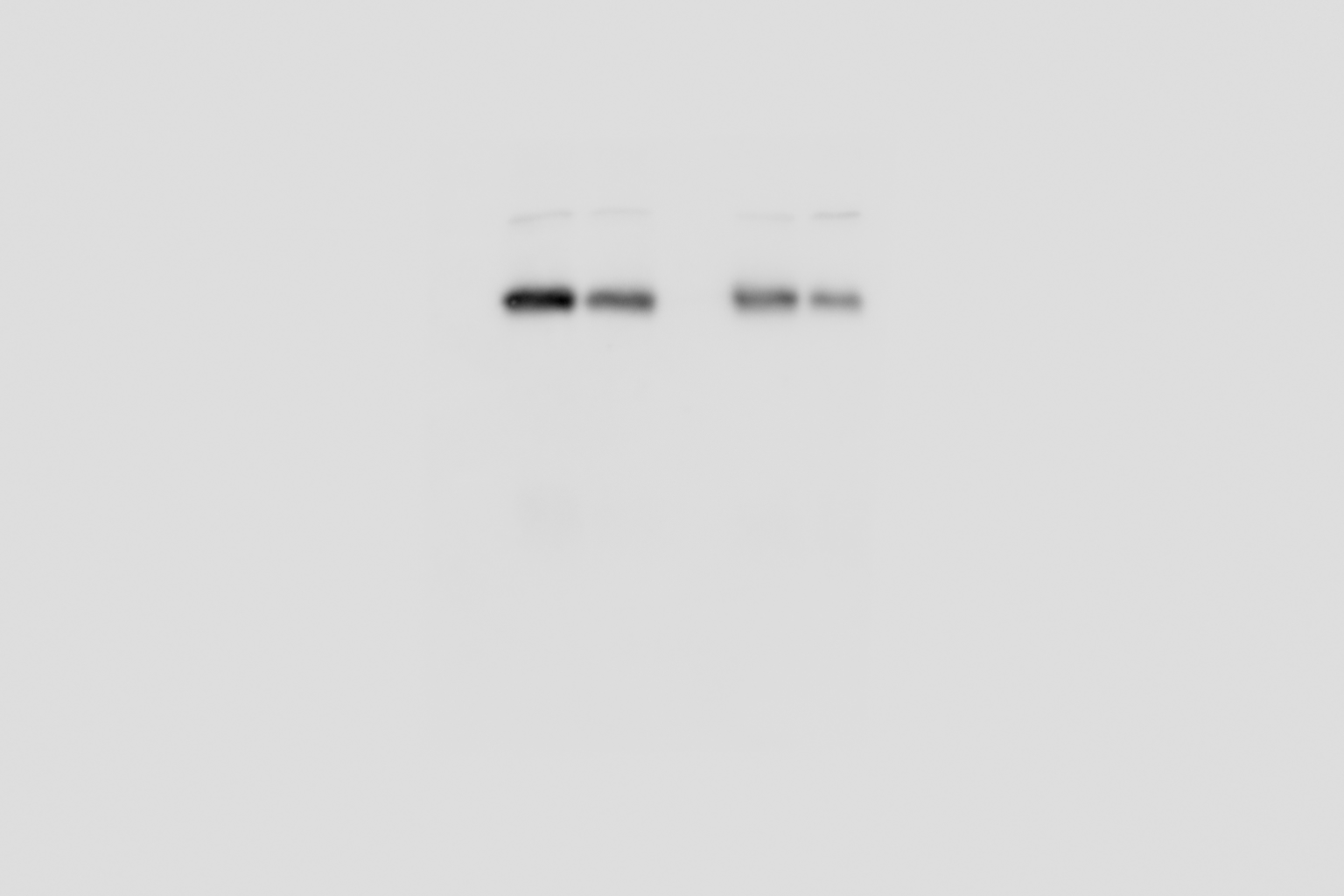

Supplement: Figure 6—source data 1. [file elife-102794-fig6-data1.zip › original images/Figure 6C anti-claudin-1 total.tif]

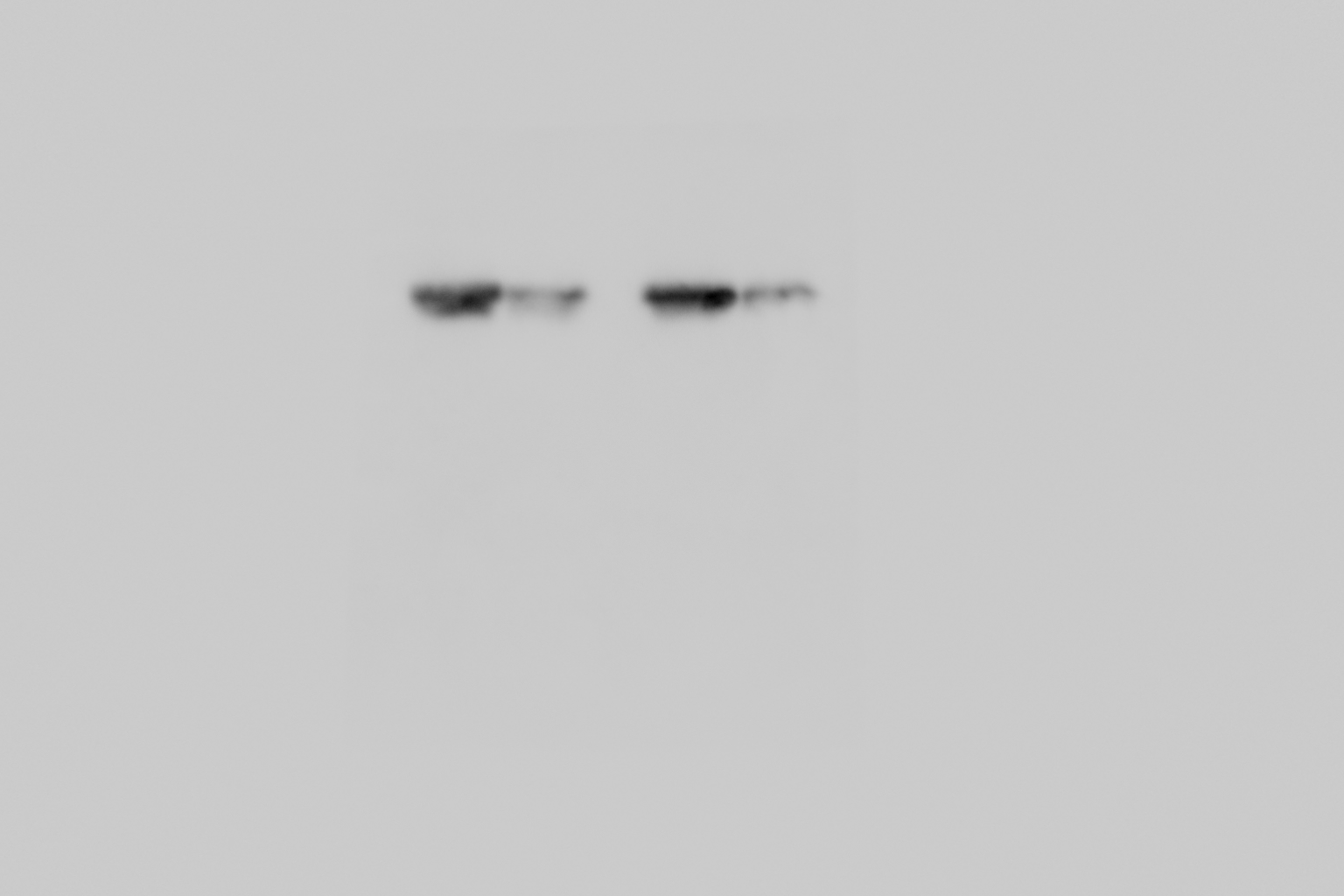

Supplement: Figure 6—source data 1. [file elife-102794-fig6-data1.zip › original images/Figure 6C anti-claudin-1 cell-surface.tif]

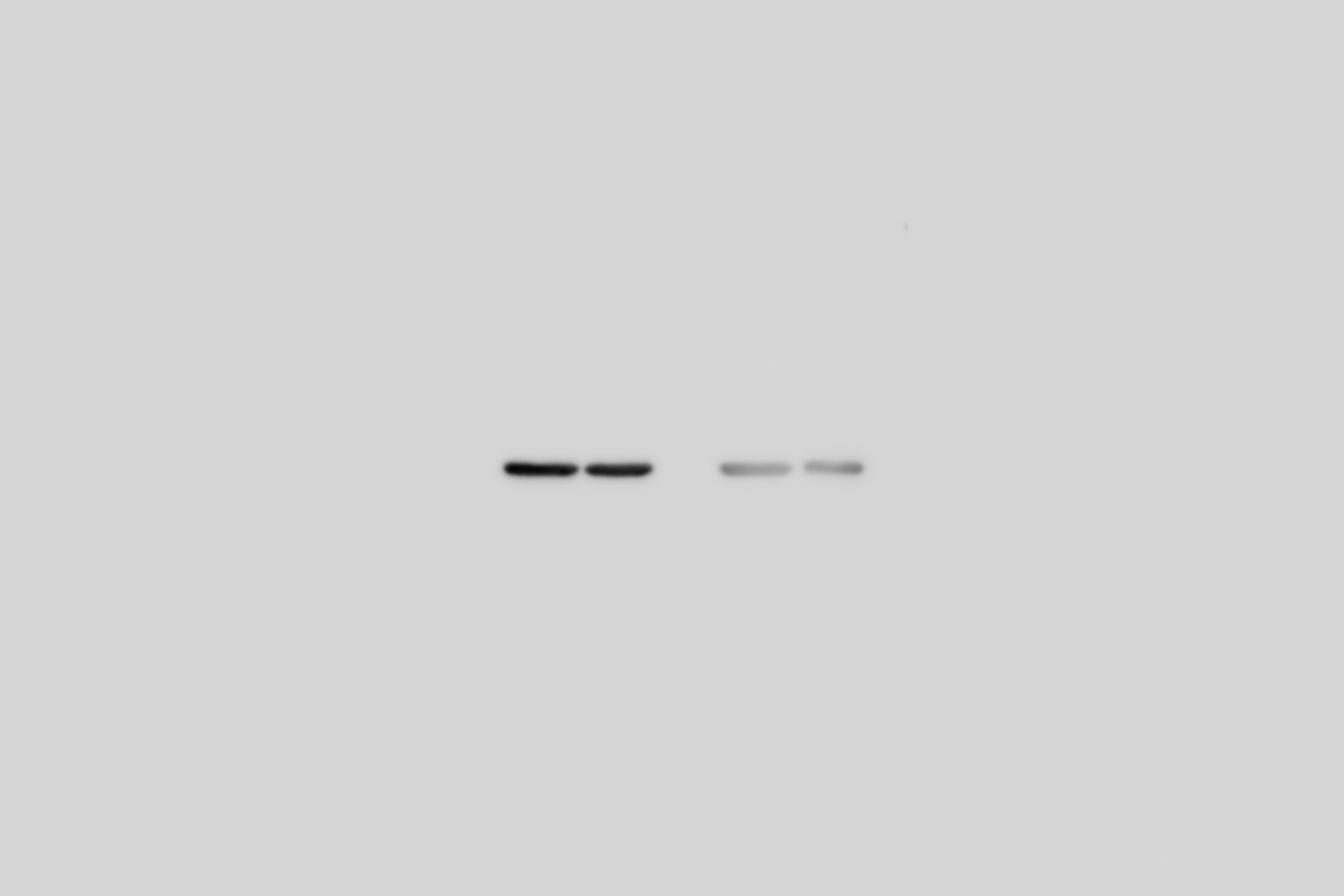

Supplement: Figure 6—source data 1. [file elife-102794-fig6-data1.zip › original images/Figure 6C anti-alpha tubulin.tif]

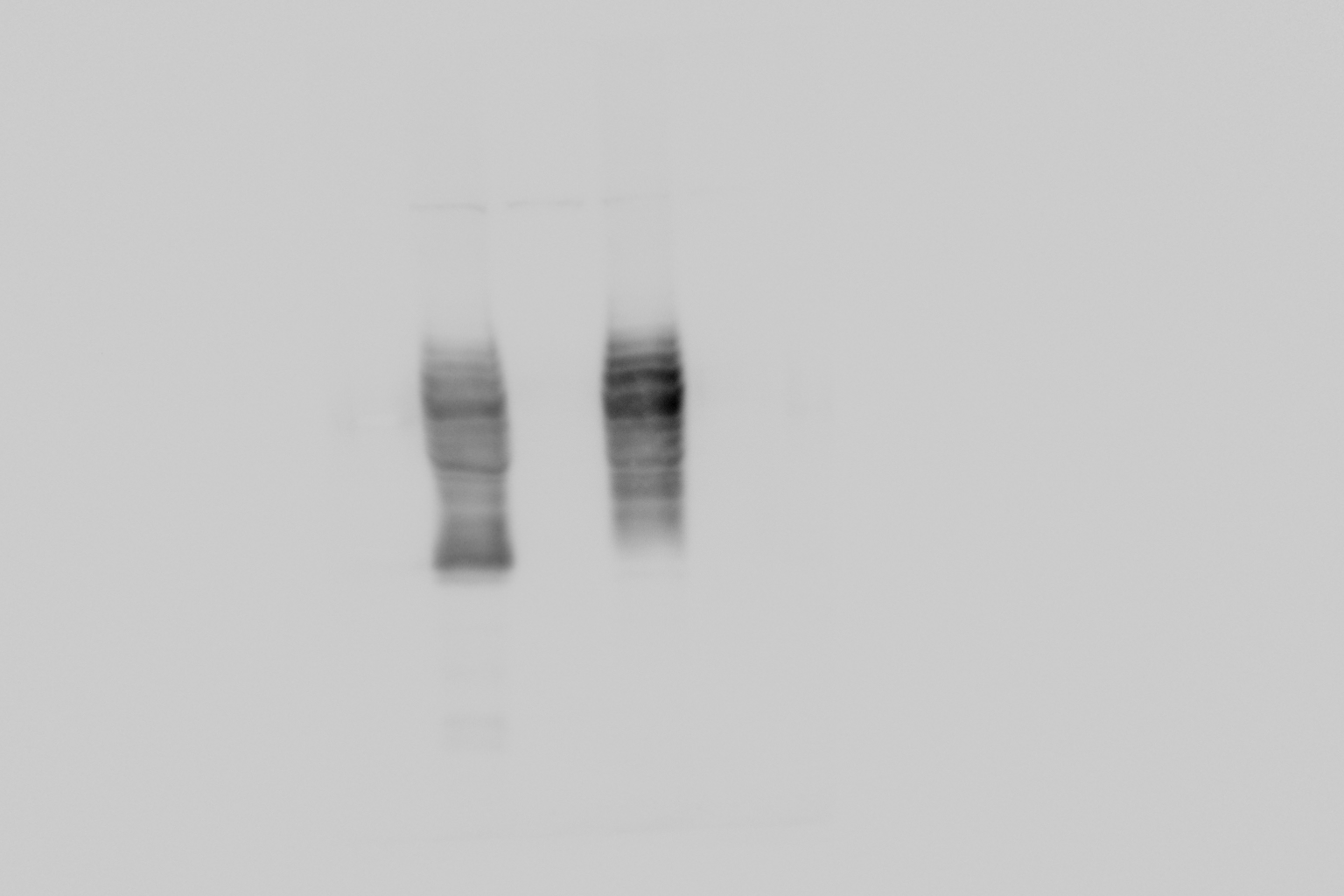

Supplement: Figure 6—source data 1. [file elife-102794-fig6-data1.zip › original images/Figure 6A anti-TROP2.tif]

# Figure6A and 6C

anti-TROP2

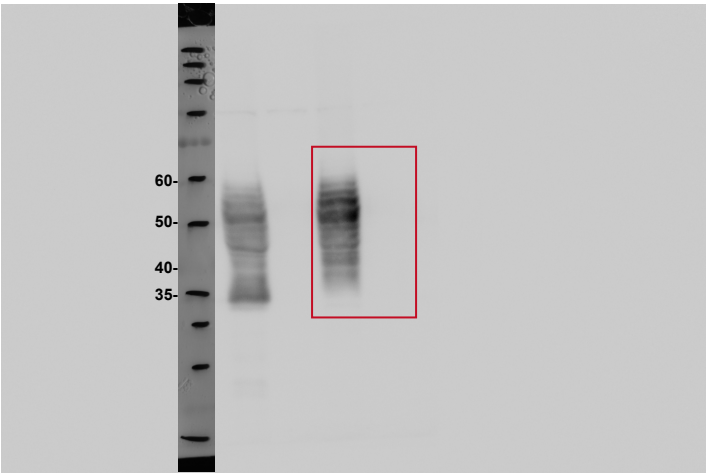

anti-claudin-1

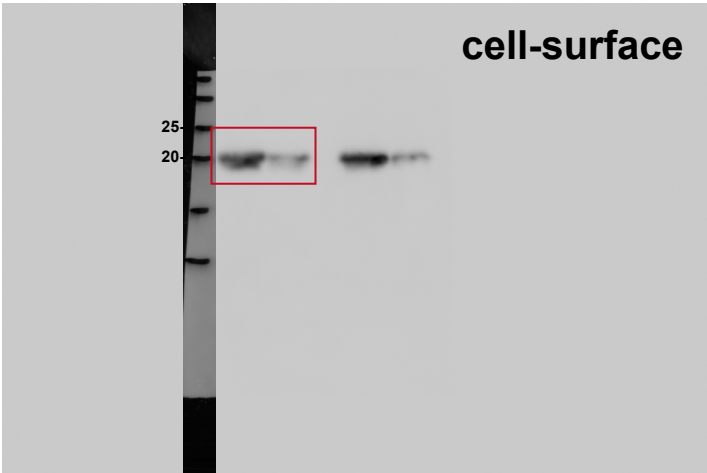

anti-claudin-1

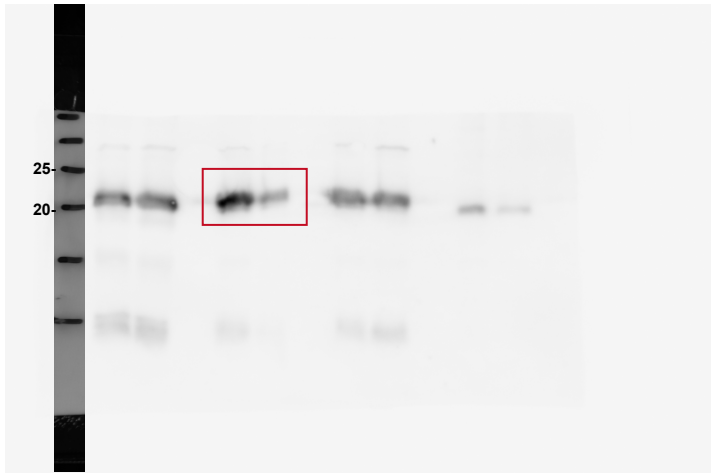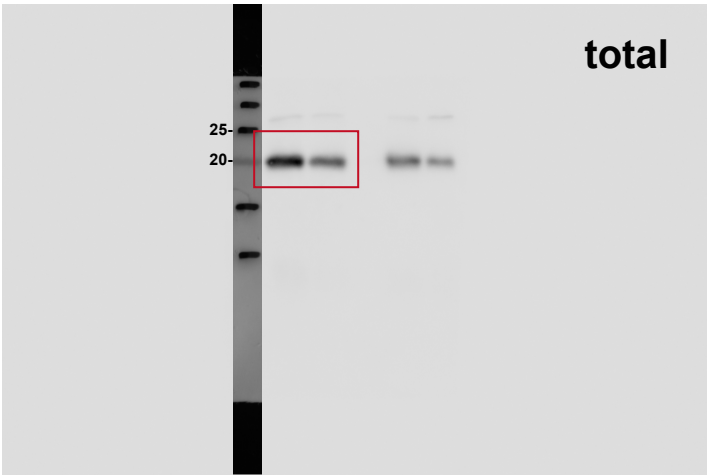

anti- $\alpha$ -tubulin

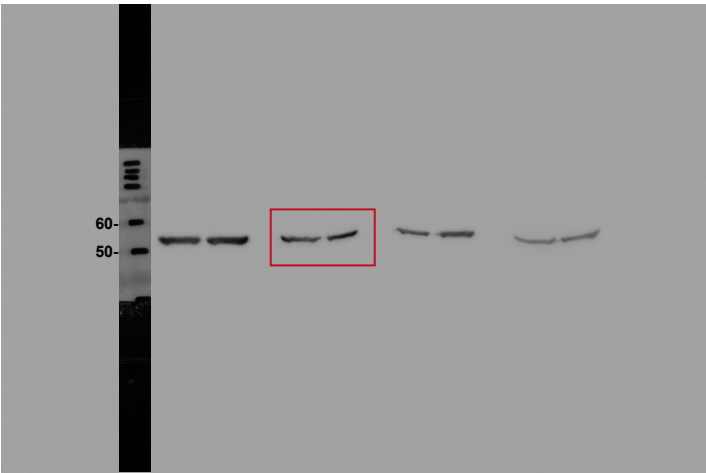

anti- $\alpha$ -tubulin

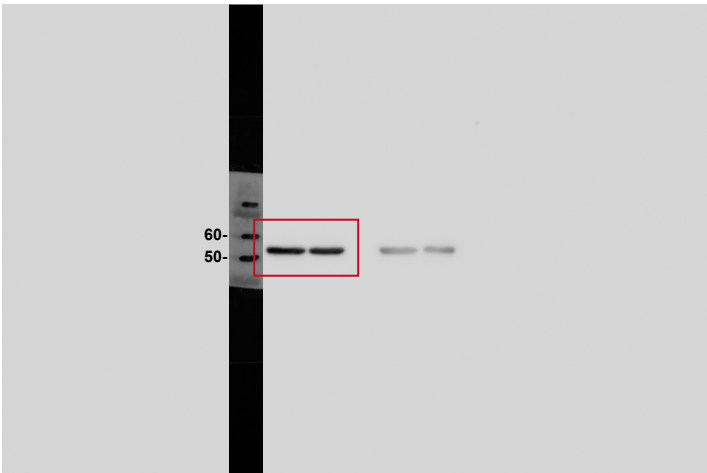

Supplement: Figure 6—source data 2. [file elife-102794-fig6-data2.pdf]

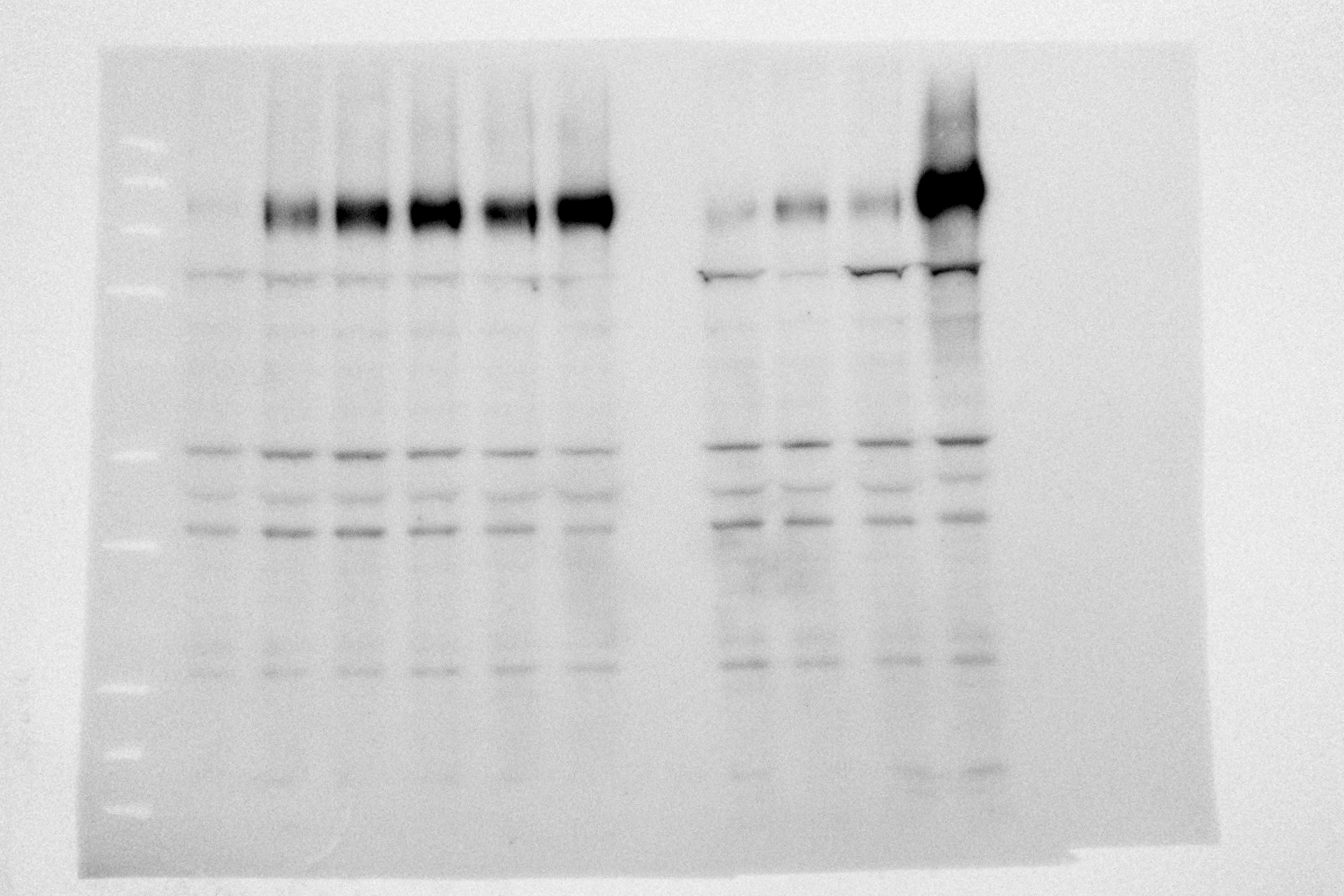

Supplement: Figure 7—source data 1. [file elife-102794-fig7-data1.zip › original images/Figure 7A anti-matriptase M69.tif]

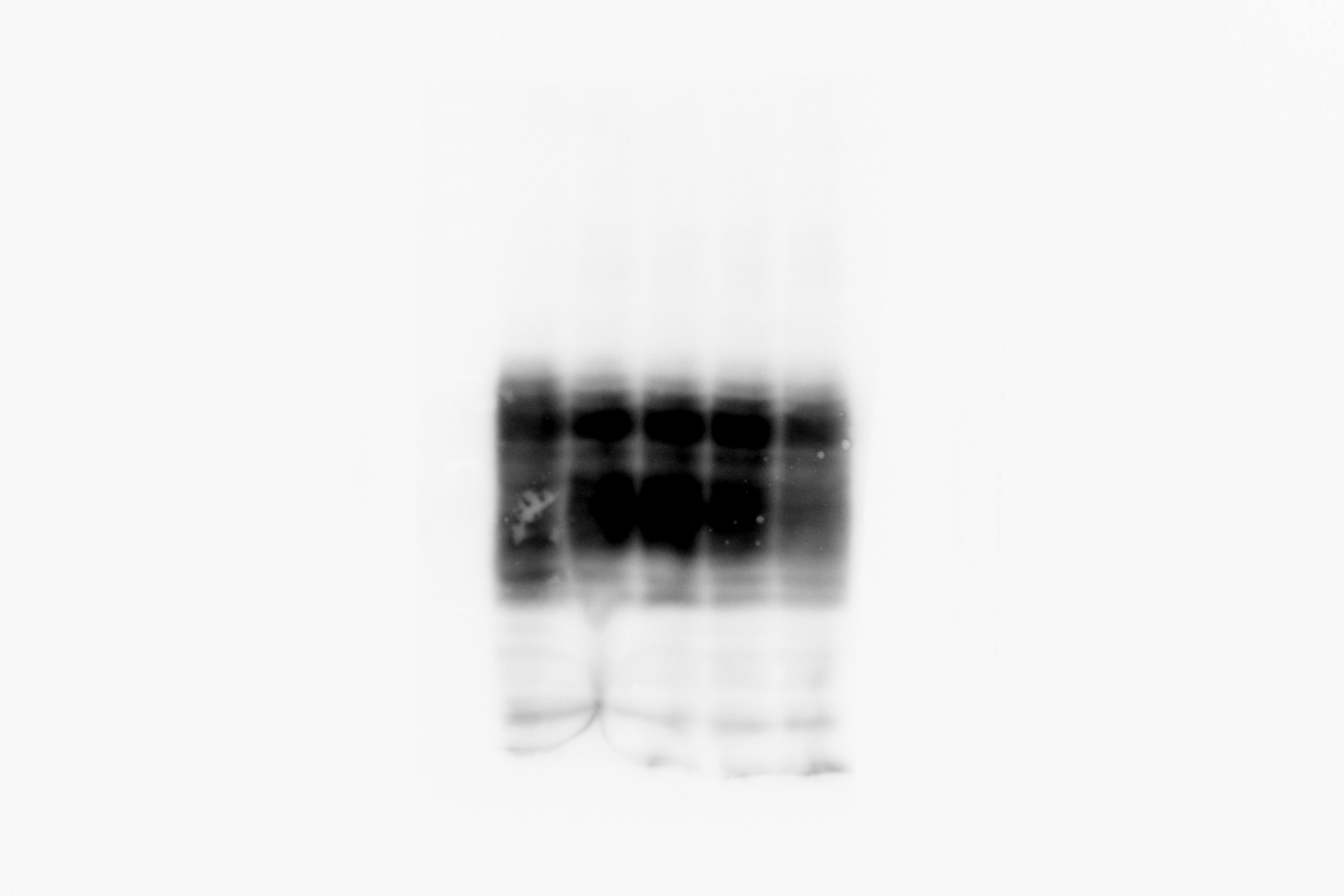

Supplement: Figure 7—source data 1. [file elife-102794-fig7-data1.zip › original images/Figure 7A anti-TROP2.tif]

# Figure7A

anti-Matriptase (M69)

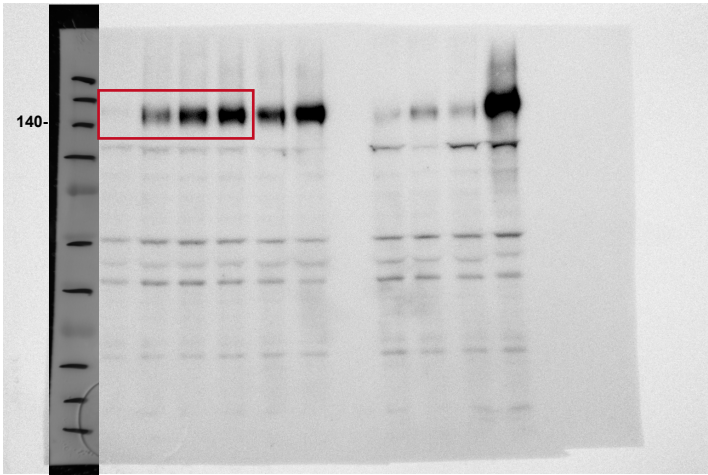

anti-Matriptase (M24)

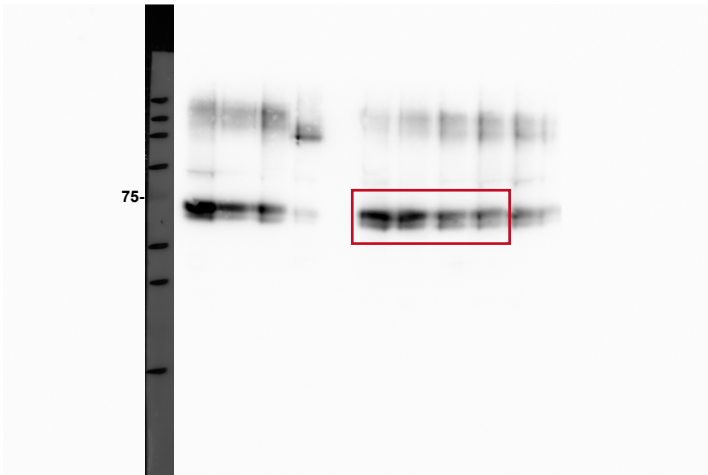

anti-TROP2

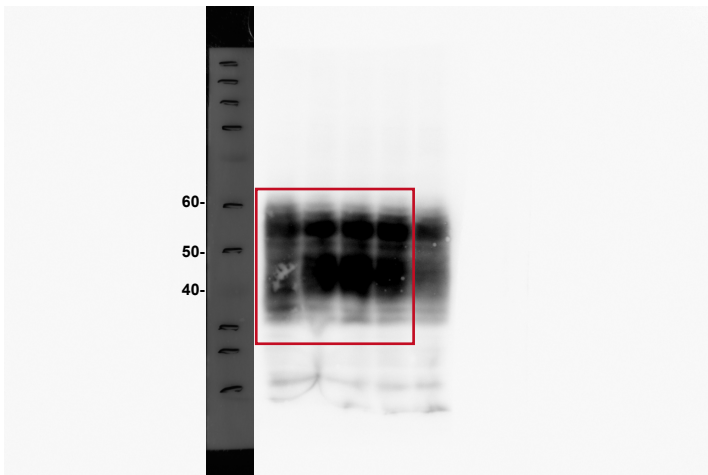

Supplement: Figure 7—source data 2. [file elife-102794-fig7-data2.pdf]

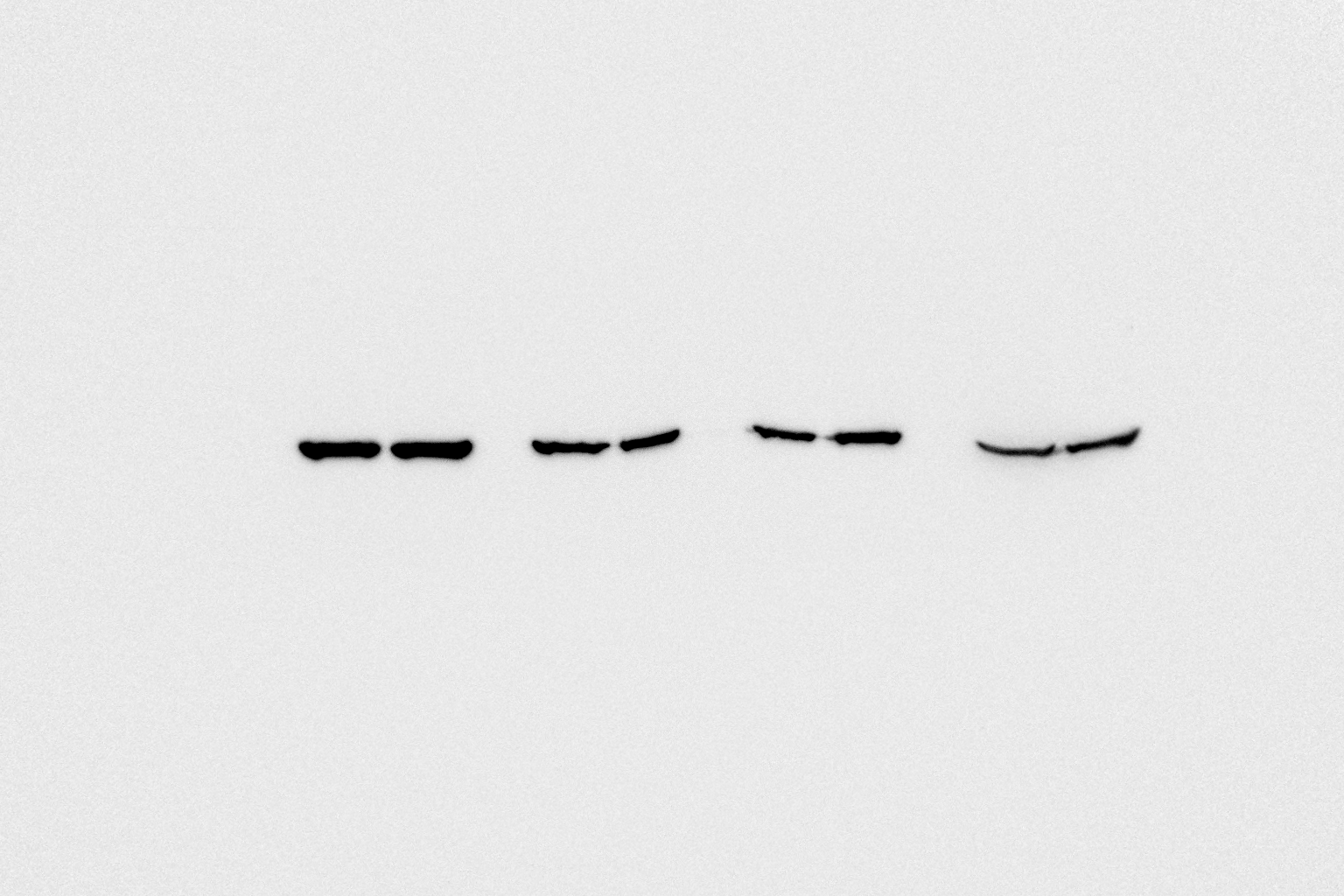

Supplement: Figure 8—source data 1. [file elife-102794-fig8-data1.zip › original images/Figure 8A anti-alpha tubulin.tif]

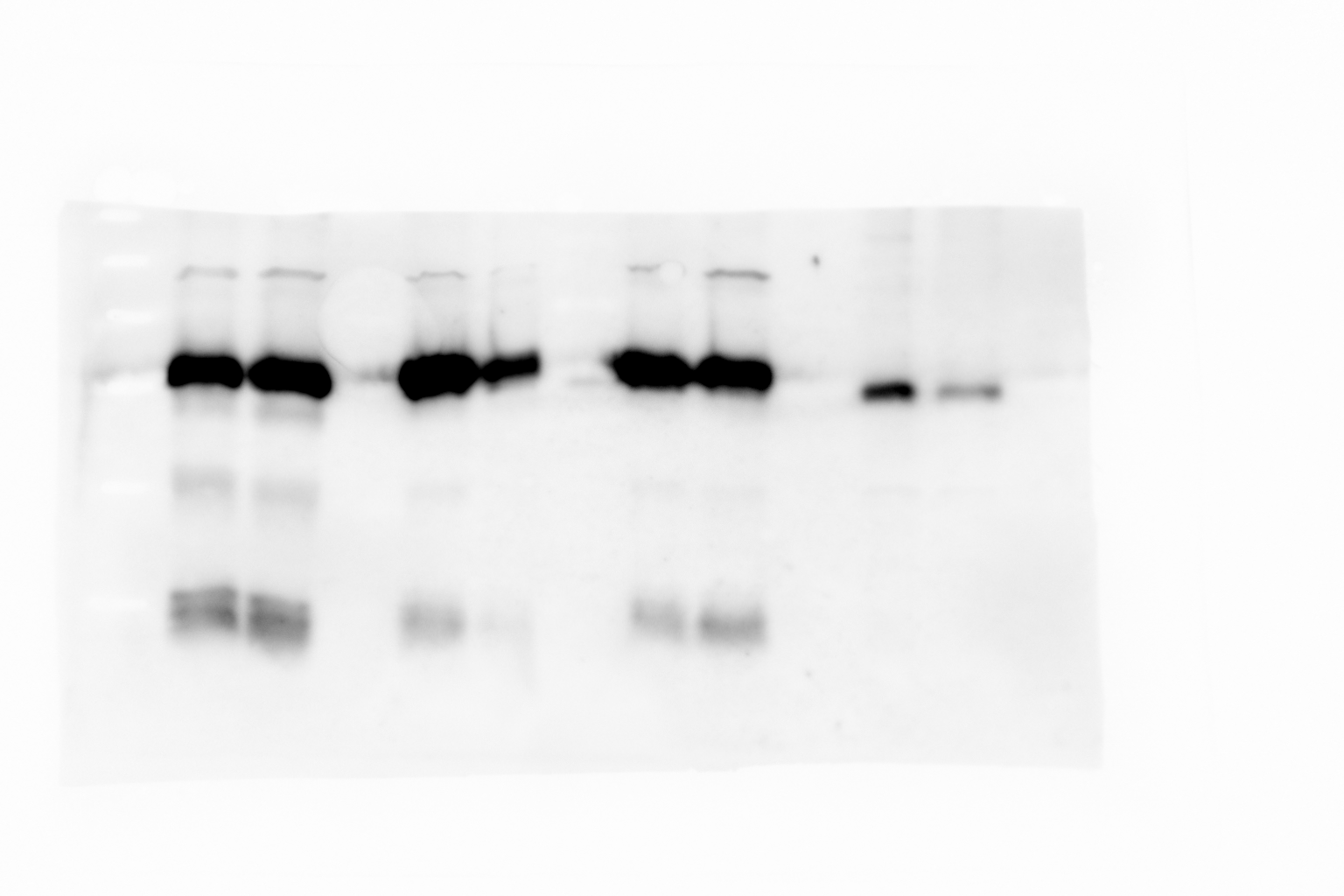

Supplement: Figure 8—source data 1. [file elife-102794-fig8-data1.zip › original images/Figure 8A anti-claudin-1.tif]

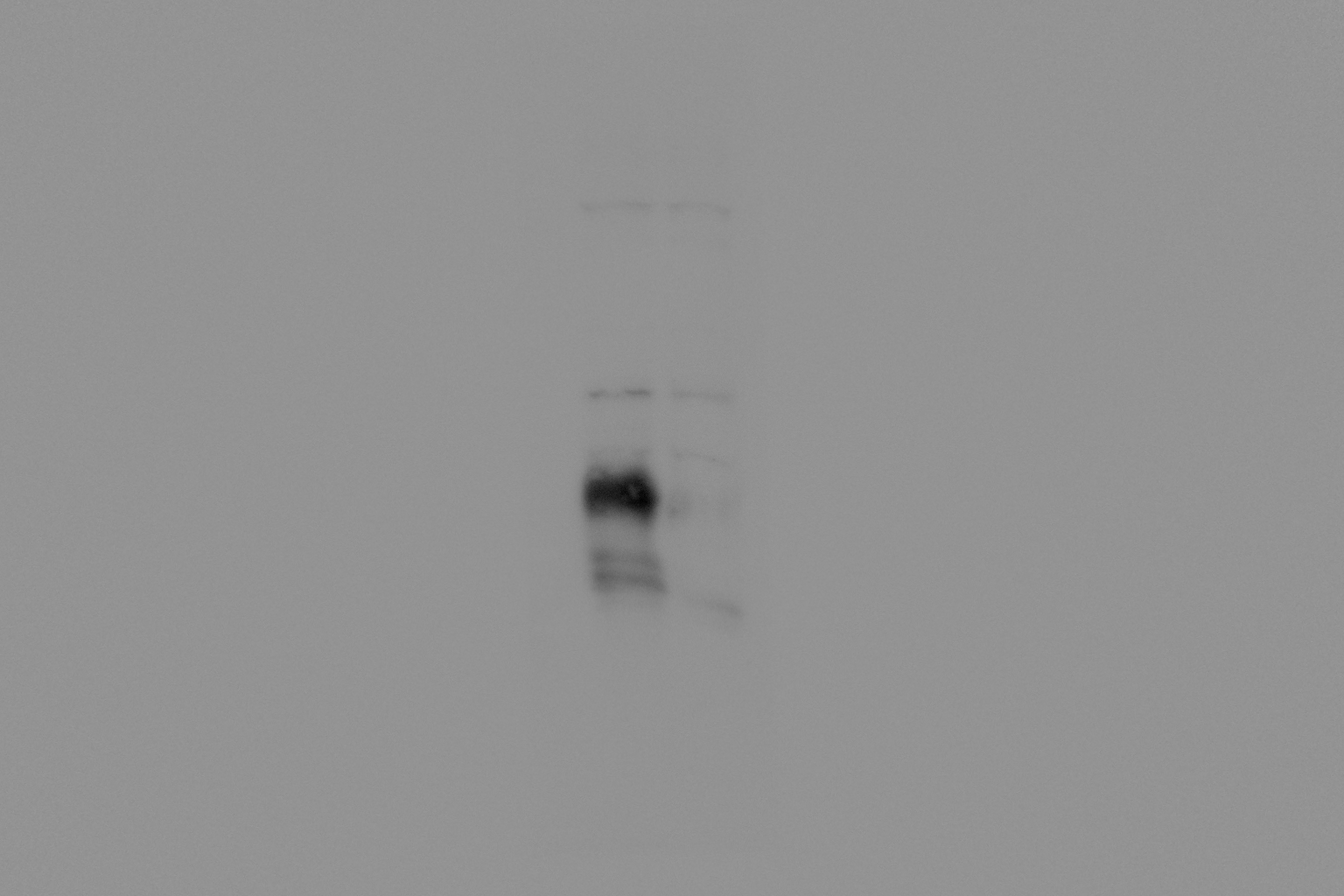

Supplement: Figure 8—source data 1. [file elife-102794-fig8-data1.zip › original images/Figure 8A anti-EpCAM.tif]

# Figure8A

anti-EpCAM

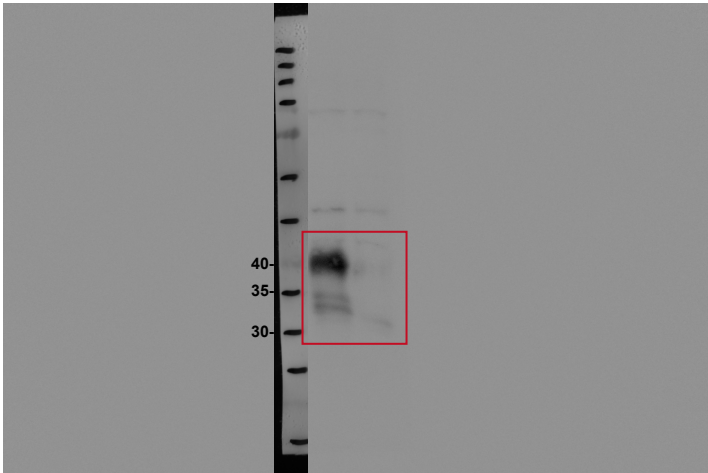

anti-claudin-1

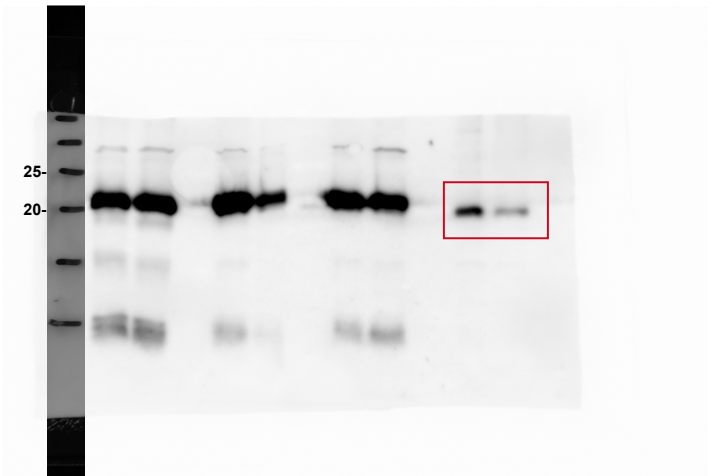

anti- $\alpha$ -tubulin

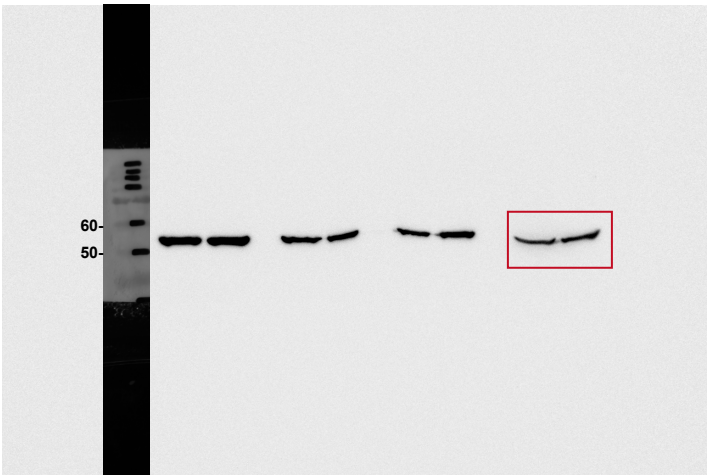

Supplement: Figure 8—source data 2. [file elife-102794-fig8-data2.pdf]
